# Analysis of long non-coding RNA RMRP in the diagnosis and prognosis of coronary artery disease

**DOI:** 10.1186/s13019-024-02870-0

**Published:** 2024-06-21

**Authors:** Haiyan Xiao, Jun Pu, Gaxue Jiang, Chenliang Pan, Jizhe Xu, Bo Zhang, Ming Bai

**Affiliations:** 1 Cardiovascular Department, Changde First Hospital of Traditional Chinese Medicine, Hunan, 415000 China; 2 Department of Cardiology, Affiliated Hospital of North Sichuan Medical College, No.1, Maoyuan South Road, Shunqing District, Nanchong, Sichuan 637000 China; 3 Heart Center, The First Hospital of Lanzhou University, Chengguan District, No.1 Donggang West Road, Lanzhou, Gansu 730000 China

**Keywords:** Long non-coding RNA, RMRP, Coronary artery disease, SYNTAX score

## Abstract

**Background:**

Long non-coding RNAs (lncRNAs) are abundant and closely related to the occurrence and development of human diseases. LncRNAs are known to play a key role in many cardiovascular diseases. The purpose of this study was to investigate the effect of the RNA component of mitochondrial RNA-processing endoribonuclease (RMRP) on the degree of coronary artery lesions and prognosis in patients with coronary artery disease (CAD).

**Methods:**

Patients who underwent coronary angiography (CAG) and dynamical-single photon emission computed tomography (D-SPECT) were selected as study subjects, and the results of CAG were reviewed, and the patients were grouped according to SYNTAX score. Evaluate the factors affecting SYNTAX scores. The follow-up analysis was conducted, and the endpoint events were major adverse cardiovascular events (MACEs). Kaplan–Meier method was used to estimate the survival rate, and multivariate Cox regression was used to analyze the relationship between RMRP and MACEs.

**Results:**

The expression level of serum RMRP in patients with CAD was significantly higher than that in healthy people. Multivariate Logistic regression analysis showed that age, low-density lipoprotein cholesterol (LDL-C), RMRP and rest left ventricular ejection fraction (LVEF) were independent factors that affected SYNTAX scores. There were 19 cases of MACEs in the high RMRP group and 9 cases in the low RMRP group, and there was a significant difference in the MACE free survival curve between the two groups. Multivariate Cox regression analysis showed that age, SYNTAX score, rest LVEF and RMRP were risk factors for MACEs.

**Conclusions:**

Serum RMRP is a key factor affecting the degree of coronary artery disease and prognosis in CAD patients.

## Background

Coronary artery disease (CAD) refers to the heart disease caused by insufficient blood supply due to coronary atherosclerosis, coronary artery spasm and stenosis, resulting in myocardial ischemia, hypoxia, or necrosis [1, 2]. According to the statistics of the National Cardiovascular Disease Center, CAD has become one of the diseases with the highest prevalence, disability and mortality rate of cardiovascular diseases in China [3]. At present, coronary angiography (CAG) is regarded as the gold standard in the clinical diagnosis of CAD, which can clearly show the anatomic morphological changes of coronary arteries and their branches, and is an indispensable examination before interventional therapy and coronary artery bypass grafting [4]. However, because CAG mainly reflects the morphological changes of coronary arteries, but cannot reflect the state of cardiomyocytes, distal microvascular lesions, and cardiac function, it has some limitations in guiding treatment and evaluating curative effect, so nuclide myocardial perfusion imaging (MPI) was appeared. Dynamical-single photon emission computed tomography (D-SPECT), as a new special cardiac scanning instrument, is the latest technical progress in the field of nuclear cardiology and the most suitable imaging technology for MPI [5, 6]. D-SPECT can also comprehensively analyze the heart by reconstructing three-dimensional map, bull's-eye map, left ventricular function parameters, which can vividly, intuitively, and accurately reflect the location and size of myocardial defect, semi-quantitatively evaluate the range of myocardial ischemia or infarction, and observe the staged ventricular wall motion, so it has obvious advantages over other instruments [7]. All the above CAD diagnostic methods have their own advantages, but they also have their own disadvantages, for example, CAG is an invasive test, while D-SPECT is expensive and low in popularity. Therefore, searching for biomarkers for disease diagnosis has always been the goal of researchers.

Long non-coding RNA (lncRNA) is a kind of RNA molecules that do not encode proteins and whose transcript length is more than 200nt [8]. LncRNA can regulate gene expression at multiple levels (epigenetic regulation, transcriptional regulation, and post-transcriptional regulation, etc.). More and more studies have shown that it can regulate the occurrence and development of various diseases, mainly involving tumors, neurodegenerative diseases, autoimmune diseases, and cardiovascular diseases [9–11]. LncRNA RNA component of mitochondrial RNA-processing endoribonuclease (RMRP) is the first RNA molecule synthesized in the nucleus and redirected to the mitochondria, which plays a role in the development of human gastric cancer, glioma, and lung cancer [12]. A recent study showed that the expression of RMRP in coronary atherosclerosis increased [13]. Another study reported that the RMRP expression in patients with CAD was upregulated [14]. Combined with these known data, it can be inferred that RMRP may interact with the occurrence and development of atherosclerotic diseases. Presently, the influence of RMRP on the diagnosis and long-term prognosis of patients with CAD is unclear, and there are also great difference in the existing studies.

In this study, the clinical indicators, cardiac function and RMRP level were analyzed to evaluate the diagnostic and prognostic efficacy of RMRP in patients with CAD, and the possible factors affecting the degree of coronary artery lesion were further analyzed.

## Methods

### Study population and sample collection

This study retrospectively analyzed 392 patients with CAD who were admitted to the First Hospital of Lanzhou University from May 2018 to December 2018. All patients with CAD underwent coronary arteriography (CAG) and echocardiogram. Exclusion criteria were as follows: 1) Previous CAD history, defined as previous percutaneous coronary intervention, coronary artery bypass grafting or myocardial infarction; 2) Acute heart failure; 3) Valvular heart disease requiring surgical treatment; 4) Severe liver and kidney diseases; 5) Patients whose life expectancy is less than one year; 6) Patients with missing clinical information. Finally, 208 CAD patients were included in this study, and all of them were diagnosed as acute coronary syndrome by angiography. Basic information, medical history and clinical indicators of all patients were recorded in this study. In addition, 141 healthy people with matching gender and age who were examined in the physical examination department at the same period were selected as the control group. This study protocol has been approved by the Ethics Committee of The First Hospital of Lanzhou University.

After enrollment, general information such as gender, age, body mass index (BMI), systolic blood pressure (SBP), diastolic blood pressure (DBP), total cholesterol (TC), triglyceride (TG), low-density lipoprotein cholesterol (LDL-C), high-density lipoprotein cholesterol (HDL-C) and fasting blood glucose (FBG) were collected. Fasting venous blood of the subjects was collected for biochemical indicators and lncRNA RMRP detection.

### SYNTAX score

According to the angiographic results of the patients, the SYNTAX score was calculated through the website. The system mainly includes the degree of coronary artery stenosis, the type of bifurcation lesions, the location and length of lesions, and whether there is thrombus. The evaluation process was interpreted independently by two experienced physicians. If the results were consistent, the data were entered; if the opinions were inconsistent, the third physician was added to discuss the results together. The physicians were blinded for the patient's other test results.

### Detection of cardiac function in patients with CAD

D-SPECT was used to assess the cardiac function of the patients. A one-day resting-stress ^99m^Tc-MIBI-resting imaging was performed, and β-blockers, theophylline, and ACEI drugs were stopped for at least 12 h before the examination. The patient was first given intravenous injection of ^99m^Tc-MIBI (9 MBq/kg), and then had a fat meal 30 min later to eliminate intestinal interference. One hour after injection of ^99m^Tc-MIBI, resting MPI was performed using D-SPECT single photon emission computed tomography (CT) from SPECTRUM, Israel. Each probe array rotates independently by 110° along its long axis, and data are collected from the patient’s sitting and supine positions, focusing on a pre-specified region of interest (ROI), including the heart. A 30–60 s pre-scan is performed before collection to define the ROI. 30 min after resting imaging, ATP drug load test was performed. Adenosine was injected through the intravenous channel at a constant rate of 140 μg/(kg·min). At the same time, ^99m^Tc-MIBI was injected at the end of adenosine infusion for 3 min, and then adenosine infusion was continued. Abnormal heart rate, blood pressure, electrocardiogram and other clinical manifestations were monitored and recorded before injection, after 3 min of injection, the end of injection and 3 min after the end of injection ATP. After the drug load test, MPI was carried out in the same way. After data collection is completed, QPS/QGS software is used for reconstruction processing, and short axis, vertical long axis and horizontal long axis tomography images are obtained, as well as left ventricular ejection fraction (LVEF), end diastolic volume (EDV), left ventricular end systolic volume (ESV) and other cardiac function parameters. Termination criteria: (1) severe angina pectoris, dyspnea and other symptoms; (2) Electrocardiograph (ECG): ST segment depression ≥ 2 mV or arch elevation ≥ 1 mV, severe malignant arrhythmia; (3) Heart rate reaches target heart rate (190—age); (4) systolic blood pressure ≥ 180 mmHg and/or diastolic blood pressure ≥ 100 mmHg; (5) Contraction pressure drop (reduction ≥ 20 mmHg, or contraction pressure < 85 mmHg) accompanied by dizziness, evil heart, sweating.

Evaluation of ATP loading ^99m^Tc-MIBI gated myocardial D-SPECT imaging: Myocardial nuclide distribution in each segment was evaluated by two or more experienced nuclear medicine physicians. On the ^99m^Tc-MIBI D-SPECT image, the left ventricular brachy-axis image was divided into the middle part, the apex and the base part, and the apex of the vertical long axis image was divided into 17 segments. The images obtained after the rest and load tests were compared. If a segment of the myocardia showed sparse or defective developer distribution during the load imaging, and then filled with developer in the rest imaging, the segment of the myocardia was considered as reversible ischemia. If resting imaging shows that the myocardia defect is less than that under load, it is considered as a partially reversible defect. If there is no obvious change in resting imaging, it is thought to be a fixation defect of the myocardium. D-SPECT imaging is positive only when blood perfusion defects are seen at least three consecutive levels.

### Reverse transcription-quantitative polymerase chain reaction (RT-qPCR)

Total RNA was extracted from serum using TRIzol, and then the concentration and purity of RNA were determined by ultraviolet spectrophotometer. Further, the total RNA was reverse-transcribed into cDNA using PrimeScript 1st Strand cDNA synthesis kit (Takara, Japan). The cDNA was amplified by PCR and the expression level of RMRP was detected. GADPH was proposed as the internal reference. The primer sequences were as follows: RMRP: (forward primer): 5'-ACTCCAAAGTCCGCCAAGA-3', (reverse primer): 5'-ACTCCAAAGTCCGCCAAGA-3'. GADPH: (forward primer): 5'-GGGAGCCAAAAGGGTCAT-3', (reverse primer): 5'-GAGTCCTTCCACGATACCAA-3'. The relative expression of RMRP was analyzed by 2^−△△Ct^ method.

### Follow-up analysis

In this study, follow-up data were collected after D-SPECT data collection began. All patients were followed up out of hospital, with the longest follow-up of 60 months and average follow-up time was 37.58 ± 14.92 months. Follow-up was conducted by trained cardiologists. Patients were followed up by phone, outpatient service, and medical records. The main endpoint events were major adverse cardiovascular events (MACEs), including cardiac death, nonfatal myocardial infarction, ischemic stroke and hospitalization for heart failure.

### Statistical analysis

SPSS 16.0 software was used for statistical analysis. Continuous variables are represented as mean ± standard deviation (SD), and categorical variables are expressed as absolute numbers. The comparison between two independent samples was conducted by t test or Chi-square test. Receiver operator characteristic (ROC) curve was constructed to analyze the clinical diagnostic value of RMRP in CAD. Pearson correlation coefficient was used to evaluate the correlation between SYNTAX score and RMRP expression level. The survival rate was estimated by Kaplan–Meier method, and the comparison between the two survival curves was performed by log-rank test. Multivariate Cox proportional hazard regression was used to analyze the relationship between RMRP and MACEs. *P* < 0.05 indicated that the difference was statistically significant.

## Results

### Comparison of baseline characteristics between control and CAD groups

The comparison of baseline data and clinical indicators between the two groups is shown in Table 1. The results showed that there were no statistically significant differences in sex ratio, age, BMI, or number of smokers between the two groups (*P* > 0.05), which indicated that the two groups were comparable. The levels of SBP, DBP, TC, TC, LDL-C and FBG in CAD group were significantly higher than those in the control group, while the levels of HDL-C were lower than that in the control group (*P* < 0.01). In addition, the D-SPECT indicators and medicine information of CAD patients can also be seen in the Table 1.

**Table 1:** Basic clinical information of the subjects

Characteristics	Healthy controls (*n* = 141)	CAD patients (*n* = 208)	*P*
Male (%)	88 (62.41)	124 (59.62%)	0.259
Age (Years)	62.36 ± 9.39	62.81 ± 9.24	0.617
BMI (kg/m^2^)	24.51 ± 4.02	24.99 ± 3.77	0.183
Current smoker (%)	53 (37.59%)	81 (38.94%)	0.894
SBP (mmHg)	130.53 ± 9.37	138.72 ± 13.91	< 0.001
DBP (mmHg)	79.26 ± 8.75	84.51 ± 7.24	< 0.001
TC (mmol/L)	4.23 ± 1.10	4.62 ± 0.94	< 0.001
TG (mmol/L)	1.35 ± 0.67	2.13 ± 0.91	< 0.001
HDL-C (mmol/L)	1.55 ± 0.34	1.32 ± 0.29	< 0.001
LDL-C (mmol/L)	2.45 ± 0.88	2.79 ± 1.02	0.009
FBG (mmol/L)	5.52 ± 1.23	5.93 ± 2.45	0.004
SYNTAX score	/	24.80 ± 10.78	/
D-SPECT characteristics			
SSS	/	3.91 ± 1.41	/
SRS	/	3.04 ± 1.15	/
SDS	/	1.93 ± 0.61	/
Stress LVEF (%)	/	59.84 ± 11.91	/
Rest LVEF (%)	/	61.17 ± 12.79	/
Medication (n, %)			
β-receptor blockers	/	106 (50.96%)	/
Statins	/	185 (88.94%)	/
Antiplatelet drugs	/	198 (95.19%)	/
ACEI/ARB	/	45 (21.63%)	/
Calcium antagonists	/	32 (15.38%)	/

Data are expressed as n or mean ± standard deviation (SD). *P* < 0.05 was a significant difference

*Abbreviations*: *BMI* body mass index, *PCI* percutaneous coronary intervention, *SBP* systolic blood pressure, *DBP* diastolic blood pressure, *TC* total cholesterol, *TG* triglyceride, *HDL-C* high-density lipoprotein cholesterol, *LDL-C* low density lipoprotein cholesterol, *FBG* fasting blood-glucose, *SSS* summed stress score, *SRS* summed rest score, *SDS* summed difference score, *LVEF* left ventricular ejection fraction

According to the results of CAG, coronary artery lesions were scored using SYNTAX scoring system. The higher the score is, the more complicated the lesions were. 208 CAD patients were divided into two groups: Mild disease group (SYNTAX score ≤ 22, *n* = 110), moderate/severe disease group (SYNTAX score > 22, *n* = 98). As shown in Table 2, the levels of age, DBP, TC and LDL-C in moderate/severe disease group were significantly higher than those in mild disease group (*P* < 0.05). In terms of cardiac function, the summed stress score (SSS) level in the moderate/severe disease group was higher than that in the mild disease group, while the levels of stress LVEF and rest LVEF were significantly lower than that in the mild disease group (*P* < 0.05).

**Table 2:** Basic clinical information of the subjects

Characteristics	SYNTAX score ≤ 22 (*n* = 110)	SYNTAX score > 22 (*n* = 98)	*P*
Male (%)	61 (55.4%)	63 (64.3%)	0.124
Age (Years)	61.57 ± 8.29	64.20 ± 10.06	0.041
BMI (kg/m^2^)	25.02 ± 4.11	24.97 ± 3.36	0.941
Current smoker (%)	39 (35.5%)	42 (42.9%)	0.171
SBP (mmHg)	137.61 ± 12.05	139.97 ± 15.72	0.223
DBP (mmHg)	83.20 ± 7.16	86.03 ± 7.07	0.005
TC (mmol/L)	4.41 ± 0.68	4.85 ± 1.13	0.001
TG (mmol/L)	2.05 ± 0.90	2.21 ± 0.93	0.184
HDL-C (mmol/L)	1.32 ± 0.23	1.33 ± 0.34	0.777
LDL-C (mmol/L)	2.51 ± 0.97	2.99 ± 1.06	0.009
FBG (mmol/L)	5.72 ± 2.33	6.16 ± 2.57	0.197
D-SPECT characteristics			
SSS	3.71 ± 1.46	4.14 ± 1.31	0.026
SRS	2.96 ± 1.24	3.13 ± 1.04	0.280
SDS	1.92 ± 0.63	1.95 ± 0.60	0.747
Stress LVEF (%)	63.29 ± 11.01	55.96 ± 11.74	< 0.001
Rest LVEF (%)	65.03 ± 10.58	56.83 ± 13.69	< 0.001

Data are expressed as n or mean ± standard deviation (SD). *P* < 0.05 was a significant difference

*Abbreviations*: *BMI* body mass index, *PCI* percutaneous coronary intervention, *SBP* systolic blood pressure, *DBP* diastolic blood pressure, *TC* total cholesterol, *TG* triglyceride, *HDL-C* high-density lipoprotein cholesterol, *LDL-C* low density lipoprotein cholesterol, *FBG* fasting blood-glucose, *SSS* summed stress score, *SRS* summed rest score, *SDS* summed difference score, *LVEF* left ventricular ejection fraction

### The expression level of serum RMRP and its value in clinical diagnosis of CAD

**Fig. 1:**
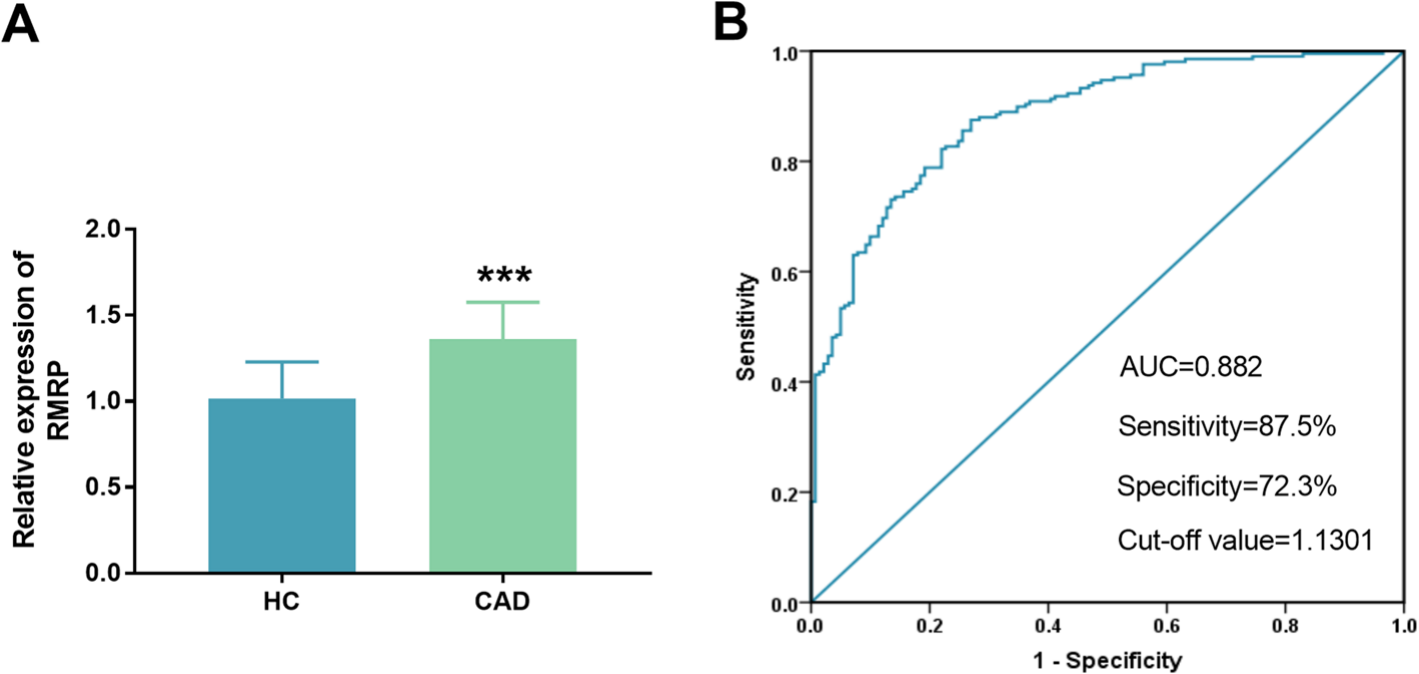
The expression level and diagnostic value of RMRP in coronary artery disease (CAD). **A** RMRP level was upregulated in CAD group compared with healthy control group. **B** ROC curve was constructed to evaluate the diagnostic value of RMRP in CAD. Area under the curve (AUC) = 0.882, Sensitivity = 87.5%, Specificity = 72.3%

As shown in Fig. 1A, it was observed that the expression of RMRP was upregulated in CAD group compared with the healthy control group (*P* < 0.001). Subsequently, ROC curve was constructed to evaluate the diagnostic value of RMRP. As illustrated in Fig. 1B, the AUC value of the ROC curve of RMRP was 0.882, and when the cut-off value was 1.1301, the sensitivity and specificity of the curve were 87.5% and 72.3%, respectively, which revealed that RMRP had high diagnostic accuracy for the diagnosis of CAD.

### Correlation between RMRP and severity of coronary artery disease

**Fig. 2:**
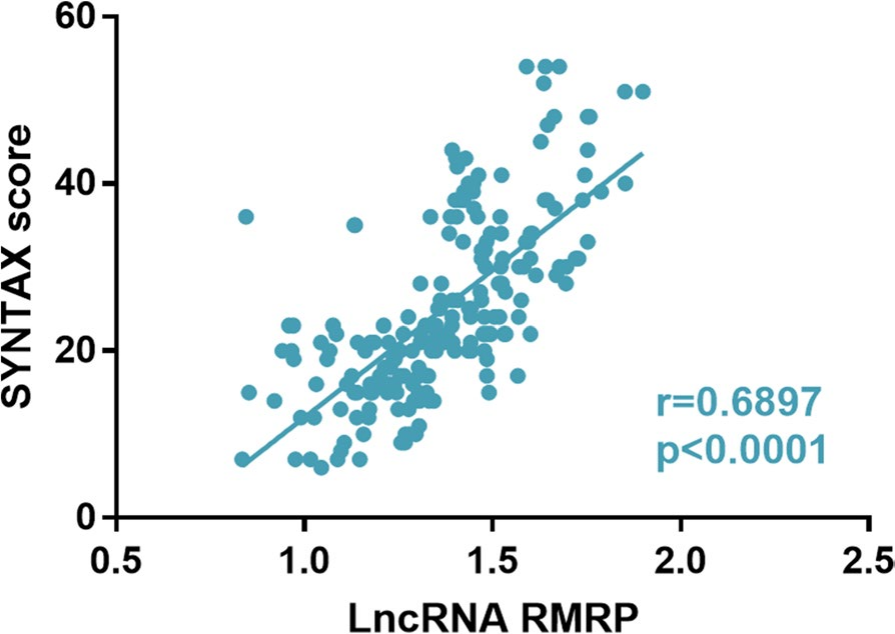
Pearson correlation analysis. SYNTAX score was positively correlated with the expression level of RMRP

Pearson correlation coefficient analysis was conducted to assess the correlation between SYNTAX scores and RMRP expression levels. The results showed that SYNTAX scores were positively correlated with the expression level of RMRP (Fig. 2, *r* = 0.6897, *P* < 0.001).

### The influencing factors of progression from mild disease to moderate/severe disease

As shown in Table 3, univariate logistic regression analysis showed that DBP (OR = 2.446, 95% CI = 1.268–4.720, *P* = 0.008), TC (OR = 2.472, 95% CI = 1.288–4.746, *P* = 0.007), stress LVEF (OR = 0.332, 95% CI = 0.172–0.639, *P* = 0.001), rest LVEF (OR = 0.382, 95% CI = 0.198–0.736, *P* = 0.004) and RMRP (OR = 2.765, 95% CI = 1.498–5.106, *P* = 0.004) were the influencing factors in the progression from mild to moderate/severe. Factors with *P* < 0.2 in the results were included in multivariate logistic regression analysis for further analysis. As shown in Table 4, DBP (OR = 2.141, 95% CI = 1.144–4.006, *P* = 0.017), TC (OR = 2.032, 95% CI = 1.095–3.772, *P* = 0.021), stress LVEF (OR = 0.433, 95% CI = 0.235–0.799, *P* = 0.007), rest LVEF (OR = 0.472, 95% CI = 0.325–0.873, *P* = 0.010), and RMRP (OR = 2.268, 95% CI = 1.241–4.145, *P* = 0.008) are independent influencing factors for disease progression.

**Table 3:** Univariate logistic regression analysis

Items	95% CI		OR	*P*
	Lower	Upper		
Age (years)	0.819	2.966	1.559	0.176
Gender (male%)	0.616	2.303	1.191	0.603
BMI (kg/m^2^)	0.404	1.484	0.774	0.440
Current smoker (%)	0.597	2.245	1.157	0.666
SBP (mmHg)	0.768	2.731	1.448	0.253
DBP (mmHg)	1.268	4.720	2.446	0.008
TC (mmol/L)	1.288	4.746	2.472	0.007
TG (mmol/L)	0.615	2.194	1.161	0.645
HDL-C (mmol/L)	0.499	1.804	0.949	0.873
LDL-C (mmol/L)	0.859	3.090	1.629	0.135
FBG (mmol/L)	0.852	3.034	1.607	0.143
SSS	0.751	2.714	1.427	0.278
SRS	0.661	2.396	1.259	0.483
SDS	0.434	1.570	0.825	0.558
Stress LVEF (%)	0.172	0.639	0.332	0.001
Rest LVEF (%)	0.198	0.736	0.382	0.004
LncRNA RMRP	1.498	5.106	2.765	0.004

Data are expressed as n or mean ± standard deviation (SD). *P* < 0.05 was a significant difference

*Abbreviations*: *BMI* body mass index, *PCI* percutaneous coronary intervention, *SBP* systolic blood pressure, *DBP* diastolic blood pressure, *TC* total cholesterol, *TG* triglyceride, *HDL-C* high-density lipoprotein cholesterol, *LDL-C* low density lipoprotein cholesterol, *FBG* fasting blood-glucose, *SSS* summed stress score, *SRS* summed rest score, *SDS* summed difference score, *LVEF* left ventricular ejection fraction

**Table 4:** Multivariate logistic regression analysis

Items	95% CI	OR	*P*
Age (years)	1.658	0.893–3.082	0.110
DBP (mmHg)	2.141	1.144–4.006	0.017
TC (mmol/L)	2.032	1.095–3.772	0.021
LDL-C (mmol/L)	1.633	0.880–3.032	0.120
FBG (mmol/L)	1.640	0.890–3.020	0.113
Stress LVEF (%)	0.433	0.235–0.799	0.007
Rest LVEF (%)	0.472	0.325–0.873	0.010
LncRNA RMRP	2.268	1.241–4.145	0.008

*Abbreviations*: *OR* odds ratio, *DBP* diastolic blood pressure, *TC* total cholesterol, *LDL-C* low density lipoprotein cholesterol, *FBG* fasting blood glucose, *LVEF* left ventricular ejection fraction

*P* < 0.05 was defined as a statistically significant difference

### Relationship between RMRP and prognosis

**Fig. 3:**
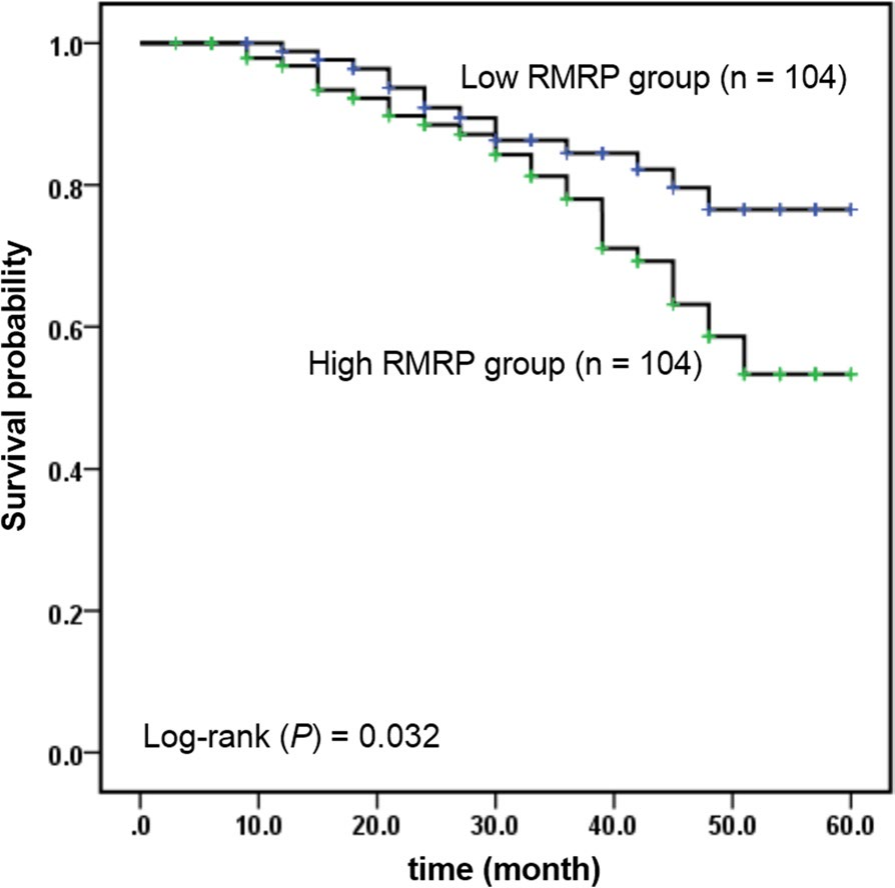
Kaplan–Meier curve. Comparison of MACEs-free survival curve between low RMRP group and high RMRP group

Patients were divided into low RMRP group (RMRP expression level < 1.3600, *n* = 104) and high RMRP group (RMRP expression level ≥ 1.3600, *n* = 104) according to the median RMRP level. The median follow-up time was 38 (5–57) months, with no loss of follow-up in either the low or high RMRP groups. There were 19 cases of MACEs in the high RMRP group, including 3 cases of cardiac death, 5 cases of non-fatal myocardial infarction, 8 cases of ischemic stroke, and 3 cases of heart failure. Furthermore, 9 cases of MACEs were observed in the low RMRP group, including 1 case of cardiac death, 3 cases of non-fatal myocardial infarction, 5 cases of ischemic stroke. There was a significant difference in survival rate between the two groups (Fig. 3, *P* = 0.032). Moreover, as shown in Table 5, multivariate Cox regression analysis showed that age (HR = 2.108, 95% CI = 1.084–4.100, *P* = 0.028), SYNTAX score (HR = 1.302, 95% CI = 1.121–4.358, *P* = 0.005), rest LVEF (HR = 0.473, 95% CI = 0.251–0.890, *P* = 0.020) and RMRP (HR = 3.623, 95% CI = 2.609–7.758, *P* < 0.001) were independent factors affecting the occurrence of MACEs.

**Table 5:** Multivariate Cox analysis for the occurrence of MACEs

Items	Multivariate analysis		
	HR	95% CI	*P*
Age	2.108	1.084–4.100	0.028
Gender	0.935	0.480–1.818	0.842
BMI	0.907	0.358–2.302	0.838
Current smoker	0.734	0.270–1.998	0.545
SBP	0.659	0.254–1.713	0.393
DBP	0.717	0.271–1.901	0.504
TC	0.789	0.352–2.014	0.065
TG	0.723	0.387–1.959	0.575
HDL-C	1.773	0.657–4.719	0.239
LDL-C	1.569	0.735–3.878	0.403
FBG	1.467	0.603–3.862	0.324
SSS	1.609	0.803–3.223	0.180
SRS	1.674	0.757–4.395	0.226
SDS	1.575	0.621–3.993	0.339
Stress LVEF	0.530	0.279–1.005	0.052
Rest LVEF	0.473	0.251–0.890	0.020
SYNTAX score	1.302	1.121–4.358	0.005
LncRNA RMRP	3.623	2.609–7.758	< 0.001

Abbreviations: *BMI* body mass index, *SBP* systolic blood pressure, *DBP* diastolic blood pressure, *TC* total cholesterol, *TG* triglyceride, *HDL-C* high-density lipoprotein cholesterol, *LDL-C* low density lipoprotein cholesterol, *FBG* fasting blood-glucose, *SSS* summed stress score, *SRS* summed rest score, *SDS* summed difference score, *LVEF* left ventricular ejection fraction

## Discussion

In recent years, with the deepening of people’s understanding of coronary heart disease and the popularization of various treatment techniques, more and more patients with CAD have been actively and effectively treated, and their prognosis has been significantly improved. Nevertheless, a great number of patients worldwide still die from cardiovascular disease every year. How to effectively prevent CAD in the early stage and reduce the occurrence of complications has become an important research topic at home and abroad. In this study, the level of RMRP was increased in the CAD group and demonstrated clinical diagnostic capability for CAD. Pearson correlation analysis showed that the expression level of RMRP was positively correlated with SYNTAX score, which indicated the degree of coronary artery lesions. Further logistic regression analysis showed that RMRP, DBP, TC, stress LVEF and rest LVEF were risk factors affecting the degree of coronary artery lesions. In addition, in the follow-up analysis, it was found that the risk of MACEs in the group with high RMRP expression was significantly higher than that in the group with low RMRP expression, and Cox regression also confirmed that age and RMRP level were the risk factors affecting the prognosis of CAD patients.

The research on the pathogenesis of RMRP and cardiovascular diseases is a hot spot at present. Chen et al. reported that RMRP was involved in the regulation of myocardial hypertrophy, and the abnormally elevated levels of RMRP were observed in hypertrophic myocardial tissue [15]. The expression of RMRP in hypoxia-induced acute myocardial infarction cells increased, and down-regulation of RMRP could significantly reduce hypoxia-induced inflammation and injury [16]. The current study found that the serum RMRP expression in patients with CAD was significantly lower than that in healthy controls group, which was consistent with previous studies. Early recognition of CAD and certain intervention can improve the prognosis of patients. Some studies have shown that lncRNA may be a good non-invasive predictor of CAD [17]. LncRNA was stored in different human fluids, including whole blood, urine, and breast milk, and it is stable under harsh conditions, such as boiling, extreme pH, and low temperature environment [18]. These characteristics make it possible to assess and monitor the pathophysiological status of patients with circulating lncRNA in clinic. Liu et al. reported that SNHG4 can promote endometrial hyperplasia by regulating miR-148a-3p, which can be used as a diagnostic biomarker and therapeutic target [19]. Wang et al. proved that the sensitivity and specificity of serum MEG3 level in detecting rectal cancer were 66.7% and 87.5%, respectively, which has the potential to be used as a biomarker for rectal cancer [20]. In this study, the AUC of the ROC curve of RMRP was 0.882, and the sensitivity and specificity were 87.5% and 72.3%, respectively, which indicated that RMRP had the advantage of high accuracy in detecting CAD.

SYNTAX score was used to evaluate the number, location, complexity and functional influence of lesions in CAG results, and to fully and comprehensively evaluated the severity of coronary artery lesions, reflecting the complexity of lesions and the technical difficulty of interventional therapy [21]. In this study, the CAD patients were divided into mild disease group (SYNTAX score ≤ 22) and moderate/severe disease group (SYNTAX score > 22) according to SYNTAX score. In the current study, compared with the mild group, patients in the moderate/severe group had higher levels of age, DBP, TC, LDL-C, and SSS, and lower levels of stress LVEF and rest LVEF. This is consistent with previous research [22]. In addition, Pearson correlation analysis showed that the SYNTAX score was positively correlated with the level of RMRP, suggesting that the degree of coronary artery disease in CAD patients may gradually deepen with the upregulation of RMRP. In subsequent logistic regression, it was found that high RMRP level was an independent risk factor for high SYNTAX score.

Due to the complexity of the SYNTAX score, its clinical operability is not good, so the value of RMRP may be more obvious for hospitals with relatively backward conditions. Here, in the follow-up study, it was found that the MACEs-free survival rate in the low RMRP group was significantly lower than that in the high RMRP group, which suggested that RMRP is an indicator affecting the long-term prognosis of patients. Further Cox regression analysis showed that RMRP, age, SYNTAX score, and rest LVEF were the independent factor affecting the occurrence of MACEs. The increase of age and SYNTAX score and the decrease of resting LVEF are recognized as three indicators affecting the prognosis of patients with CAD [23, 24]. There are few studies on the prognosis of RMRP in cardiovascular disease, but in some cancer studies, high expression of RMRP is a risk factor for poor prognosis in cancer patients [25]. However, with the further study of RMRP and the pathogenesis of cardiovascular diseases, RMRP involves many aspects of cardiovascular diseases, including atherosclerosis, endothelial cell dysfunction, oxidative stress, inflammation. Therefore, the monitoring of RMRP concentration may have great potential for the diagnosis of atherosclerotic diseases. Combined with the traditional diagnosis methods of the disease, the early monitoring of RMRP is helpful to prevent the occurrence and progress of cardiovascular diseases.

Based on previous studies, this study further confirmed that the level of serum RMRP is related to the progress of CAD and can predict the occurrence of MACEs. In actual clinical work, full attention should be paid to the changes in RMRP level, so as to truly achieve early detection and intervention of CAD and complications. There are several limitations to this study. Firstly, this study is a single-center study with a small sample size, and the findings of this study often need to be further confirmed by multi-center trials. Secondly, this study was a retrospective study, focusing on patients who received CAG and D-SPECT, and it is unclear whether the results of this study are applicable to those patients who were not included and did not receive D-SPECT. Thirdly, we only assessed the baseline serum RMRP data, and did not measure the patient’s RMRP during follow-up period.

## Conclusions

In conclusion, this study shows that a high level of RMRP is significantly associated with the severity of coronary artery disease and the incidence of MACEs, which provides a simple, convenient, and operable detection index for the clinical diagnosis, treatment, and prognosis of CAD, and provides an exact clinical basis for the prevention and treatment of CAD.

## Data Availability

Datasets used and/or analyzed for this study are available from the corresponding author upon appropriate request.

## Abbreviations

lncRNA: Long non-coding RNAs
RMRP: RNA component of mitochondrial RNA-processing endoribonuclease
D-SPECT: Dynamical-single photon emission computed tomography
CAD: Coronary artery disease
CAG: Coronary angiography
MACEs: Major adverse cardiovascular events
MPI: Myocardial perfusion imaging
BMI: Body mass index
SBP: Systolic blood pressure
DBP: Diastolic blood pressure
TC: Total cholesterol
TG: Triglyceride
LDL-C: Low-density lipoprotein cholesterol
HDL-C: High-density lipoprotein cholesterol
FBG: Fasting blood glucose
RT-qPCR: Reverse transcription-quantitative polymerase chain reaction
CT: Computed tomography
ROI: Region of interest
EDV: End diastolic volume
ESV: End systolic volume
SD: Standard deviation
